# Hessian-Regularized Co-Training for Social Activity Recognition

**DOI:** 10.1371/journal.pone.0108474

**Published:** 2014-09-26

**Authors:** Weifeng Liu, Yang Li, Xu Lin, Dacheng Tao, Yanjiang Wang

**Affiliations:** 1 College of Information and Control Engineering, China University of Petroleum (East China), Qingdao, Shandong, China; 2 Shenzhen Institutes of Advanced Technology, Chinese Academy of Sciences, Shenzhen, Guangdong, China; 3 The Chinese University of Hong Kong, Hong Kong, China; 4 Centre for Quantum Computation and Intelligent Systems, and Faculty of Engineering and Information Technology, University of Technology, Sydney, Ultimo, New South Wales, Australia; Banner Alzheimer's Institute, United States of America

## Abstract

Co-training is a major multi-view learning paradigm that alternately trains two classifiers on two distinct views and maximizes the mutual agreement on the two-view unlabeled data. Traditional co-training algorithms usually train a learner on each view separately and then force the learners to be consistent across views. Although many co-trainings have been developed, it is quite possible that a learner will receive erroneous labels for unlabeled data when the other learner has only mediocre accuracy. This usually happens in the first rounds of co-training, when there are only a few labeled examples. As a result, co-training algorithms often have unstable performance. In this paper, Hessian-regularized co-training is proposed to overcome these limitations. Specifically, each Hessian is obtained from a particular view of examples; Hessian regularization is then integrated into the learner training process of each view by penalizing the regression function along the potential manifold. Hessian can properly exploit the local structure of the underlying data manifold. Hessian regularization significantly boosts the generalizability of a classifier, especially when there are a small number of labeled examples and a large number of unlabeled examples. To evaluate the proposed method, extensive experiments were conducted on the unstructured social activity attribute (USAA) dataset for social activity recognition. Our results demonstrate that the proposed method outperforms baseline methods, including the traditional co-training and LapCo algorithms.

## Introduction

The rapid development of Internet technology and computer hardware has resulted in an exponential increase in the quantity of data uploaded and shared on media platforms [1] [2]. Processing these data presents a major challenge to machine learning, especially since most of the data are unlabeled and are described by multiple representations in different computer vision applications [3] [4]. One of the earliest multi-view learning schemes was co-training, in which two classifiers are alternately trained on two distinct views in order to maximize the mutual agreement between the two views of unlabeled data [5]. In general, the co-training algorithms train a learner on each view separately and then force the learners to be consistent across views.

A number of co-training approaches have been proposed in many applications [6] [7] [8] [9] since the original implementation [10] [11] and can be divided into four groups: (1) co-EM [12] [13]; (2) co-regression [14] [15]; (3) co-regularization [16]; and (4) co-clustering. The co-EM algorithm combines co-training with the probabilistic EM approach by using naive Bayes as the underlying learner [12]. Brefeld and Scheffer [13] subsequently developed a co-EM version of support vector machines (SVMs). The co-regression algorithm can also be used to extend co-training to regression problems; for example, Zhou and Li [14] employed two k-nearest neighbor regressors with different distance metrics to develop a co-training style semi-supervised regression algorithm, and Brefeld et al. [15] investigated a semi-supervised least squares regression algorithm based on the co-learning schema. The co-regularization algorithm formulates co-training as a joint complexity regularization between the two hypothesis spaces, each of which contains a predictor approximating the target function [16]. The co-clustering algorithms [17] [18] [19] apply the idea of co-training to unsupervised learning settings with the assumption that a point will be assigned to the same cluster in each view by the true underlying clustering.

Although many co-training variants have been developed, most co-training-style methods aim to obtain satisfactory performance in multi-view learning by minimizing the disagreement between two classifiers. However, it is likely that a learner will receive erroneous labels on unlabeled data when the other learner has only mediocre accuracy. This usually happens in the first rounds of co-training, when there are only a few label examples.

To address the aforementioned problem, here we propose Hessian-regularized co-training, in which regularization is integrated into the learner training process of each view to significantly boost performance. Specifically, each Hessian is obtained from a particular view of examples, which is then used to penalize the classifier along the potential manifold. Comparing other manifold regularizations e.g. Laplacian regularization, Hessian has a richer nullspace and steers the learned function that varies linearly along the underlying manifold. Thus Hessians can properly exploit the local distribution geometry of the underlying data manifold [20] [21], and therefore Hessian regularization can significantly boost the generalizability of a classifier, especially when only a small number of labeled examples exist with a large number of unlabeled examples.

To evaluate the proposed Hessian regularized co-training, we conduct extensive experimentation on the unstructured social activity attribute (USAA) dataset [22] [23] for social activity recognition [24] [25] [26] [27]. The USAA dataset contains eight different semantic class videos, which are home videos of social occasions, including birthday parties, graduation parties, music performances, non-music performances, parades, wedding ceremonies, wedding dances, and wedding receptions. We compare the proposed Hessian regularized co-training (HesCo) with traditional co-training and Laplacian regularized co-training (LapCo). The experimental results demonstrate that the proposed method outperforms the baseline algorithms.

## Method Overview

In the standard co-training setting, we are given a two-view training dataset of 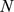 examples, including 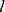 labeled examples, i.e., 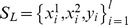, and 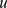 unlabeled examples, i.e., 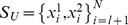, where 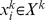 for 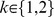 is the 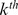 view feature vector of the 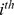 example and 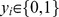 is the class label of the 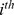 example (in the remainder of this section we use 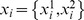 to denote the 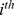 example and 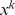 to denote the 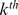 view feature). Labeled examples are drawn from 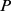 and unlabeled examples are drawn from the marginal distribution 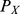 of 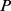, in that 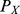 is a compact manifold 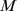. Generally, 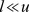. The goal of co-training is to predict the labels of unseen examples by learning a hypothesis from the training dataset.

On the other hand, manifold learning assumes that close example pairs 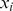 and 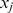 will have similar conditional distribution pairs 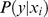 and 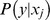 [28]. It is therefore important to properly exploit the intrinsic geometry of the manifold 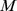 that supports 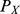, and here we employ Hessian regularization to explore the geometry of the underlying manifold. Hessian regularization penalizes the second derivative along the manifold. This approach ensures that the learner is steered linearly along the data manifold, and it is superior to first order manifold learning algorithms, including Laplacian regularization, for both classification and regression [29] [30] [31]. The effectiveness of Hessian regularization has been well explored by Eells [32], Donoho [21], and Kim [20].

For convenience, we list the important notations used in this paper in Table 1.

**Table 1:** List of important notations.

Notation	Description
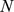	Number of training examples
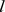	Number of labeled examples
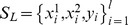	Labeled examples
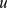	Number of unlabeled examples
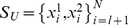	Unlabeled examples
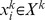	The 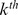 view feature vector of the 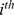 example
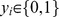	The class label of the 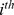 example
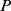	Probability of examples
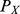	Marginal distribution of 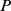
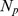	Collection of k-nearest neighbors at example 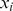
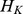	Reproducing kernel Hilbert space (RKHS)
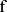	Predicted vector of training examples
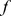	Classifier
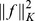	Classifier complexity penalty term
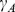	Parameter of 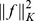
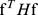	Hessian regularizer term
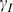	Parameter of Hessian regularizer term

In this section, we first briefly introduce Hessian regularization derived from Hessian eigenmaps [21] [20]. We then present the Hessian-regularized support vector machine (HesSVM), which is applied as the classifier for each view of co-training. Finally, we summarize the proposed Hessian regularized co-training.

### 2.1 Hessian regularization

Given a smooth manifold 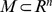 and the neighborhood 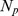 at point 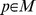, the 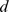 largest eigenvectors obtained by performing PCA on the points in 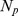 correspond to an orthogonal basis of the tangent space 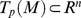 at point 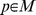. We can then define the Hessian of a function, 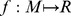, using the local coordinates. Suppose that 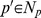 has local coordinates 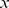. The rule 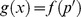 defines a function 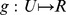 on a neighborhood of 0 in 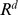. The Hessian of the function 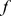 at 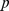 in tangent coordinates can then be defined as the ordinary Hessian of 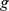 by 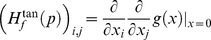. The construction of the tangent Hessian of a point depends on the choice of the coordinate system used in the underlying tangent space 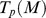. Fortunately, the usual Frobenius norm of a Hessian matrix is invariant to coordinate changes [21]. Therefore, we have the Hessian regularizer that measures the average curviness of 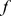 along the manifold 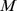 as 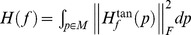, where 
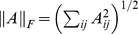
 is the usual Frobenius norm of matrix 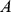.

We summarize the computation of Hessian regularization in the following steps [20] [21] [29] [30].

Step 1: Finding the *k*-nearest neighbors 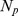 of sample 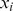 and form a matrix 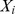 whose rows consist of the centralized examples 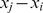 for all 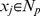.
Step 2: Estimate the orthonormal coordinate system of the tangent space 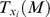 by performing a singular value decomposition of 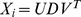.
Step 3: Performing the Gram-Schmidt orthonormalization process on the matrix 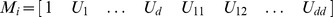 and resulting 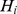. The Frobenius norm of 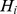 is 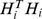.
Step 4: Summing up 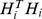 over all examples and then resulting the Hessian regularization 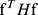.

### 2.2 The Hessian-regularized support vector machine (HesSVM)

The Hessian-regularized support vector machine (HesSVM) for binary classification takes the form of the following optimization problem:
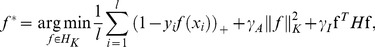
(1)where 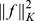 is the classifier complexity penalty term in an appropriate reproducing kernel Hilbert space (RKHS) 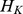, 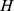 is the Hessian matrix, and the term 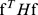 is the Hessian regularizer to penalize 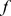 along the manifold 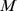. Parameters 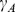 and 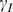 balance the loss function and the regularization terms, respectively.

According to the representer theorem [28], the solution to problem (1) is given by
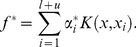
(2)


By substituting (2) back into (1) and introducing the slack variables 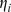 for 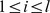, the primal problem of HesSVM is the following:
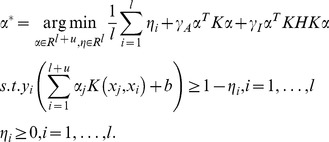
(3)


Using the Lagrangian method, the solution to (3) is
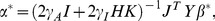
(4)where 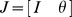 is an 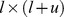 matrix with 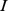 as the 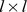 identity matrix and 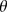 as the 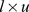 zero matrix, 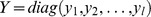, and 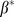 is the solution to the following problem:
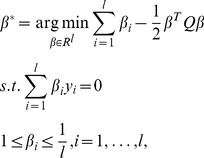
(5)where 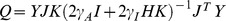, 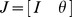 is the 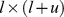 matrix, 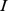 is the 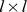 identity matrix, 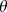 is the 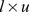 zero matrix, and 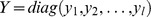.

Problem (3) can then be transformed into a standard quadratic programming problem (5) that can be solved using an SVM solver.

### 2.3 Hessian regularized co-training (HesCo)

Similar to standard co-training algorithms, HesCo also iteratively learns the classifiers from the labeled and unlabeled training examples. In each iteration, HesSVM exploits the local geometry to significantly boost the prediction of unlabeled examples, which helps to effectively augment the training set and update the classifiers. Table 2 summarizes the procedure of HesCo by integrating HesSVM into CoTrade [33].

**Table 2:** Summary of the HesCo algorithm for co-training.

Algorithm 1. HesCo algorithm for co-training
**Input:** training set 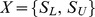 , 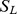 is the labeled example set and 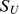 isthe unlabeled example set.
**Output:** classifier 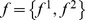
1. Calculate Hessian matrix 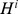 ( 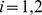 );
2. Initialize classifiers 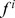 under view 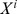 ( 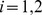 ) by HesSVM;
3. **Repeat**
4. Predict labels of unlabeled examples of each view in 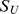 using classifiers 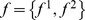 , respectively;
5. Estimate the labeling confidence of each classifier;
6. Augment the labeled example set and form a new training set, 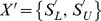
7. Update classifiers 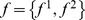 with the new training set 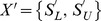 by HesSVM;
8. **Until** {specified stopping criterion is satisfied}.
9. Return 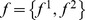

### 2.4 Complexity analysis

Suppose we are given 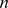 examples, the computation of the inverse of a dense Gram matrix leads to 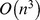 and general HesSVM implementations typically have a training time complexity that scales between 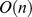 and 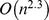. Hence in each iteration of co-training, the time cost is approximately 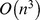. Denote the number of iteration as 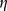, the total cost of the proposed method is about 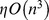.

## Experiments

We conducted experiments for social activity recognition on the USAA database [22] [23]. The USAA database is a subset of the CCV database [34] and contains eight different semantic class videos, as described above.

In our co-training experiments, we used tagging features as one view and visual features as the other. The tagging features are the 69 ground-truth attributes provided by Fu et al. [22] [23], and the visual features are low features that concatenate SIFT, STIP, and MFCC according to [34].

We used the same training/testing partition as in [22] and [23], in which the training set contains 735 videos and the testing set contains 731 videos. Each class contains around 100 videos for training and testing, respectively. In our experiments, we selected any two of the eight classes to evaluate performance, resulting in a total of 28 one vs. one binary classification experiments. We randomly divided the training set 10 times to examine the robustness of the different methods. In each experiment, we selected 10%, 20%, 30%, 40%, and 50% of the training videos as labeled examples, and the rest as unlabeled examples, for initialization assignment. Parameters 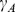 and 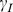 in HesSVM and LapSVM were tuned using the candidate set 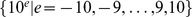. The parameter 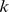, which denotes the number of neighbors when computing the Hessian and graph Laplacian, was set to 100.

We compared the proposed HesCo with CoTrade and Laplacian regularized co-training (LapCo). The accuracy and mean accuracy (MA) for all classes were used as assessment criteria.

**Figure 1:**
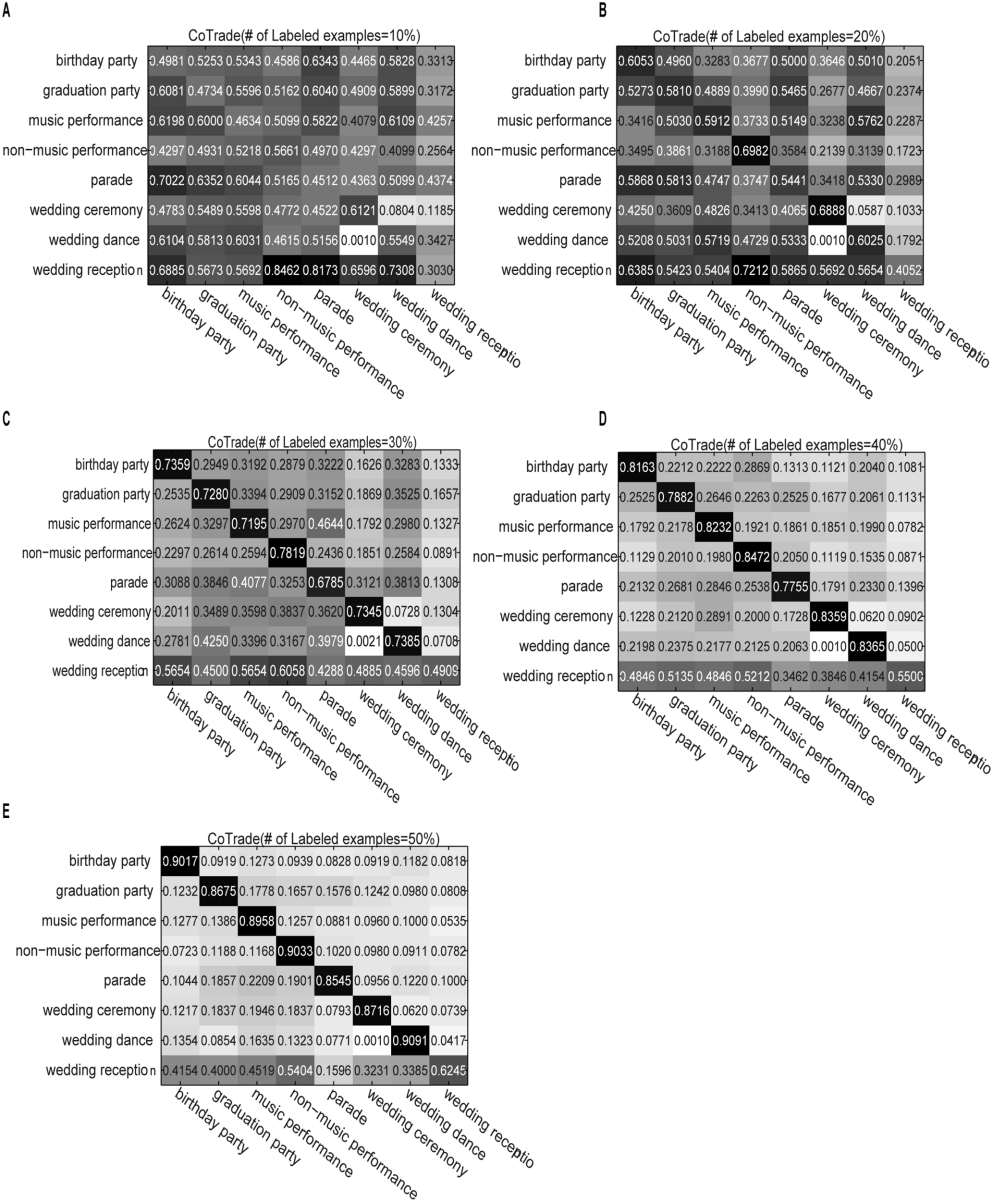
Confusion matrix for CoTrade on the eight activity classes. The subfigures correspond to the performance of the algorithm using different numbers of labeled examples. The x- and y-coordinates are the class labels. (A) Confusion matrix obtained with 10% labeled examples. (B) Confusion matrix obtained with 20% labeled examples. (C) Confusion matrix obtained with 30% labeled examples. (D) Confusion matrix obtained with 40% labeled examples. (E) Confusion matrix obtained with 50% labeled examples.

**Figure 2:**
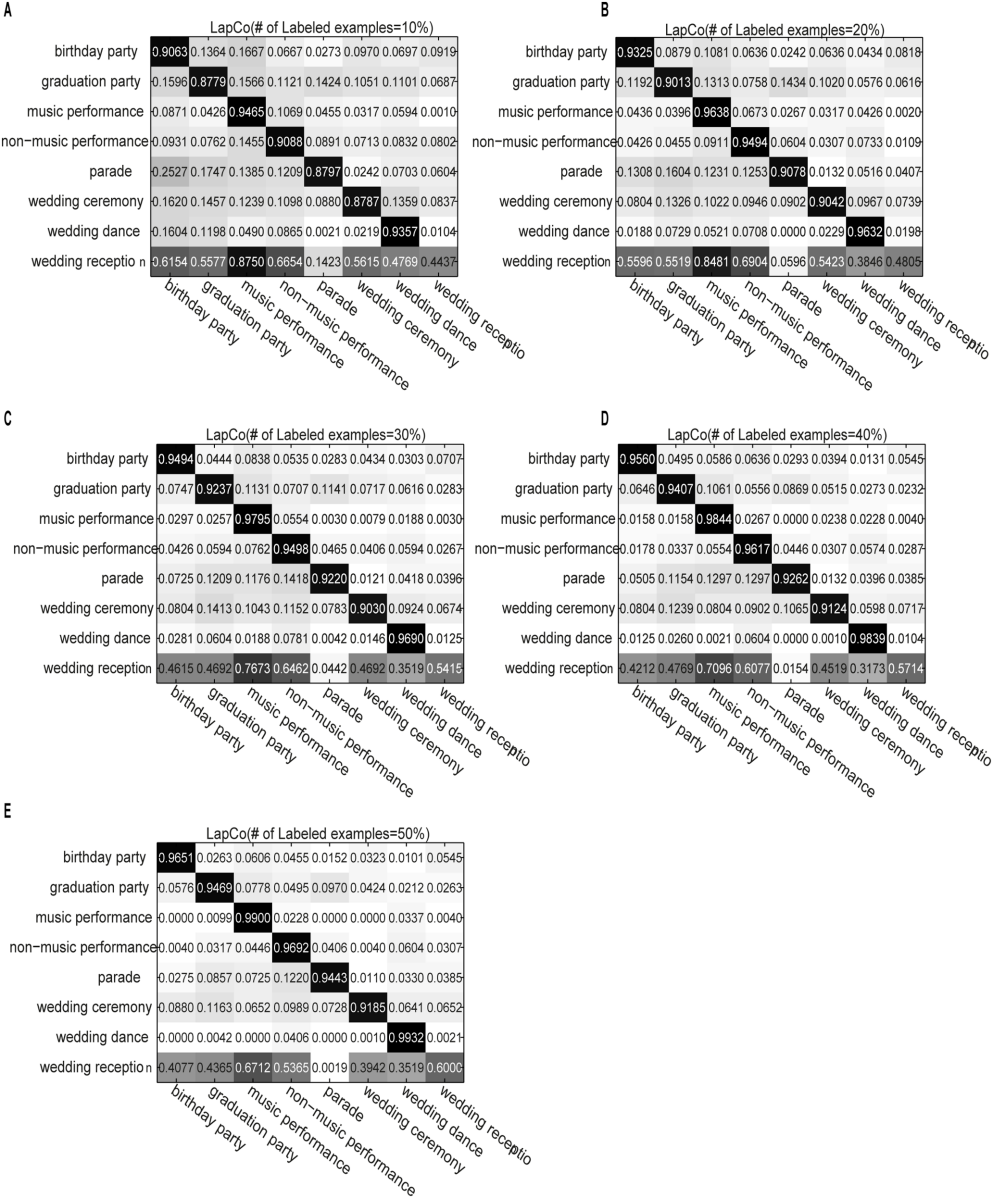
Confusion matrix for LapCo on the eight activity classes. The subfigures correspond to the performance of the algorithm using different numbers of labeled examples. The x- and y-coordinates are the class labels. (A) Confusion matrix obtained with 10% labeled examples. (B) Confusion matrix obtained with 20% labeled examples. (C) Confusion matrix obtained with 30% labeled examples. (D) Confusion matrix obtained with 40% labeled examples. (E) Confusion matrix obtained with 50% labeled examples.

**Figure 3:**
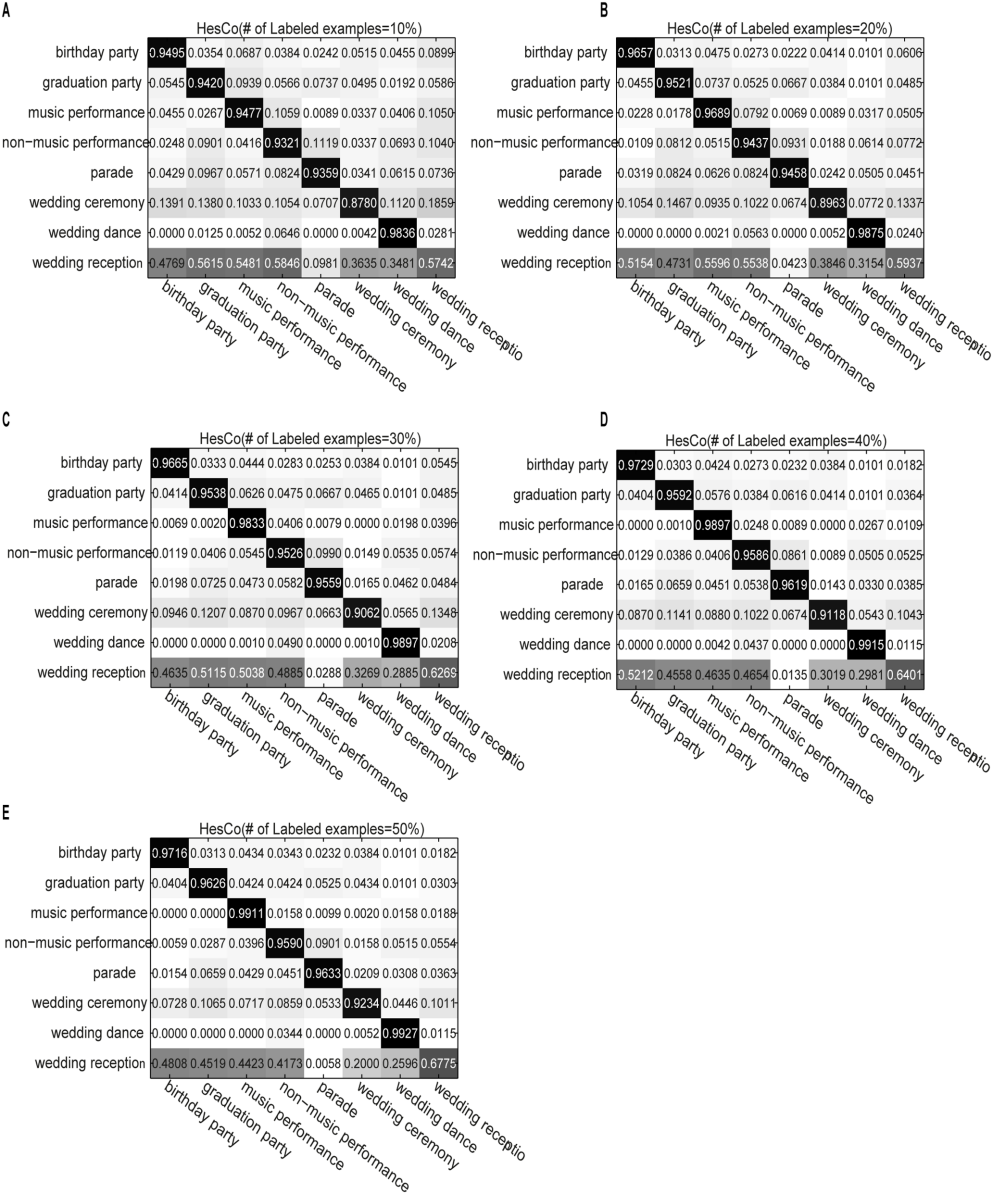
Confusion matrix for HesCo on the eight activity classes. The subfigures correspond to the performance of the algorithm using different numbers of labeled examples. The x- and y-coordinates are the class labels. (A) Confusion matrix obtained with 10% labeled examples. (B) Confusion matrix obtained with 20% labeled examples. (C) Confusion matrix obtained with 30% labeled examples. (D) Confusion matrix obtained with 40% labeled examples. (E) Confusion matrix obtained with 50% labeled examples.

Figure 1 shows the confusion matrix for the CoTrade method on the eight social activity classes. The subfigures correspond to the performance of the algorithm using different numbers of labeled examples. The x- and y-coordinates are the class labels. Figures 2 and 3 similarly demonstrate the performances of LapCo and HesCo, respectively. From Figure 1 we can see that the errors are distributed across the category labels when there are only a few labeled examples, and from Figures 2 and 3 we can see that LapCo and HesCo significantly improve performance, especially when the number of labeled examples is small.

**Figure 4:**
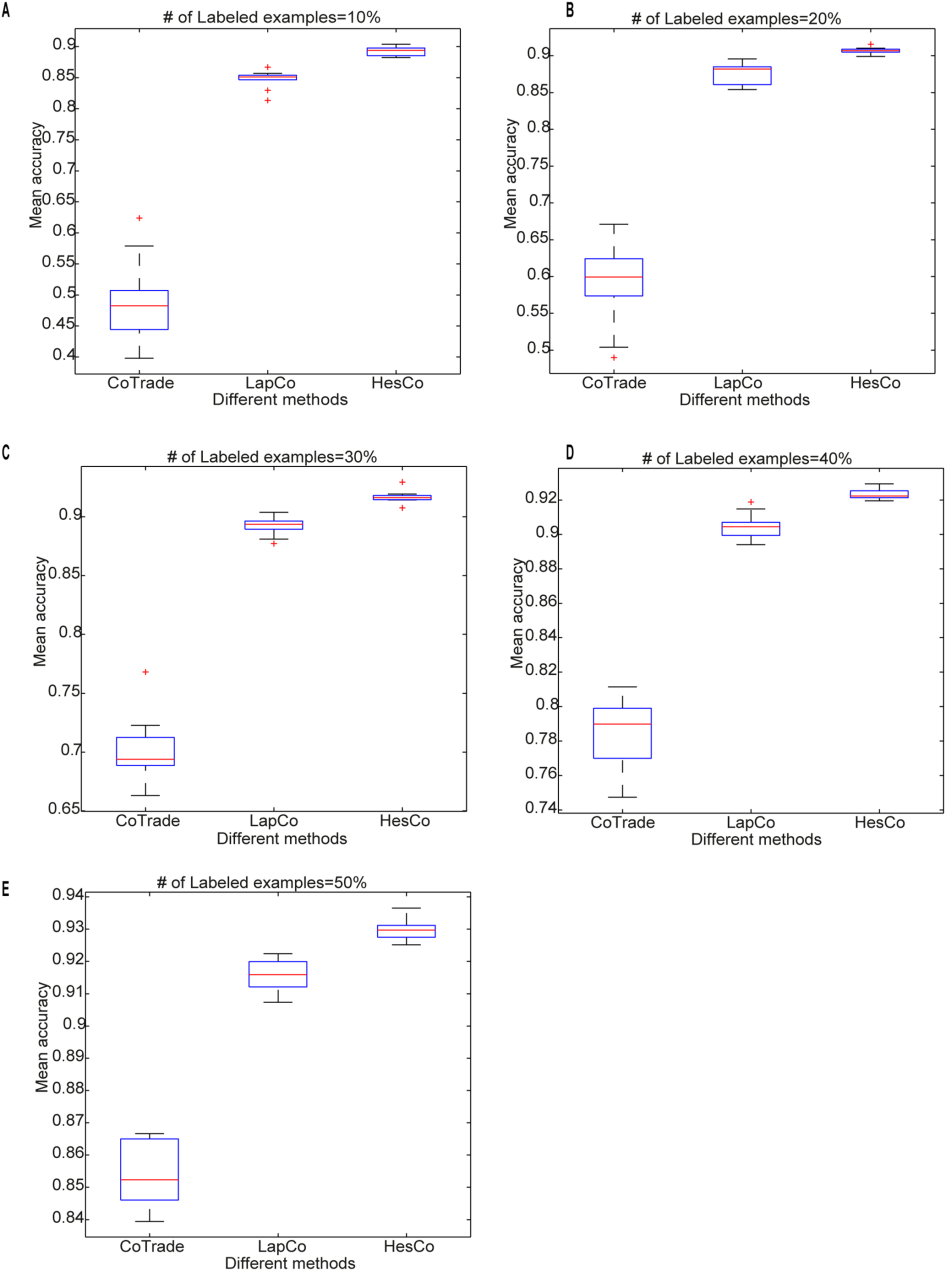
Mean accuracy (MA) boxplots for the different co-training methods. Each subfigure corresponds to one case of labeled examples. (A) MA obtained using 10% labeled examples. (B) MA obtained using 20% labeled examples. (C) MA obtained using 30% labeled examples. (D) MA obtained using 40% labeled examples. (E) MA obtained using 50% labeled examples.

Figure 4 shows the MA boxplots for the different co-training methods, with each subfigure corresponding to one case of labeled examples. LapCo and HesCo both perform better than CoTrade, and HesCo outperforms LapCo.

**Figure 5:**
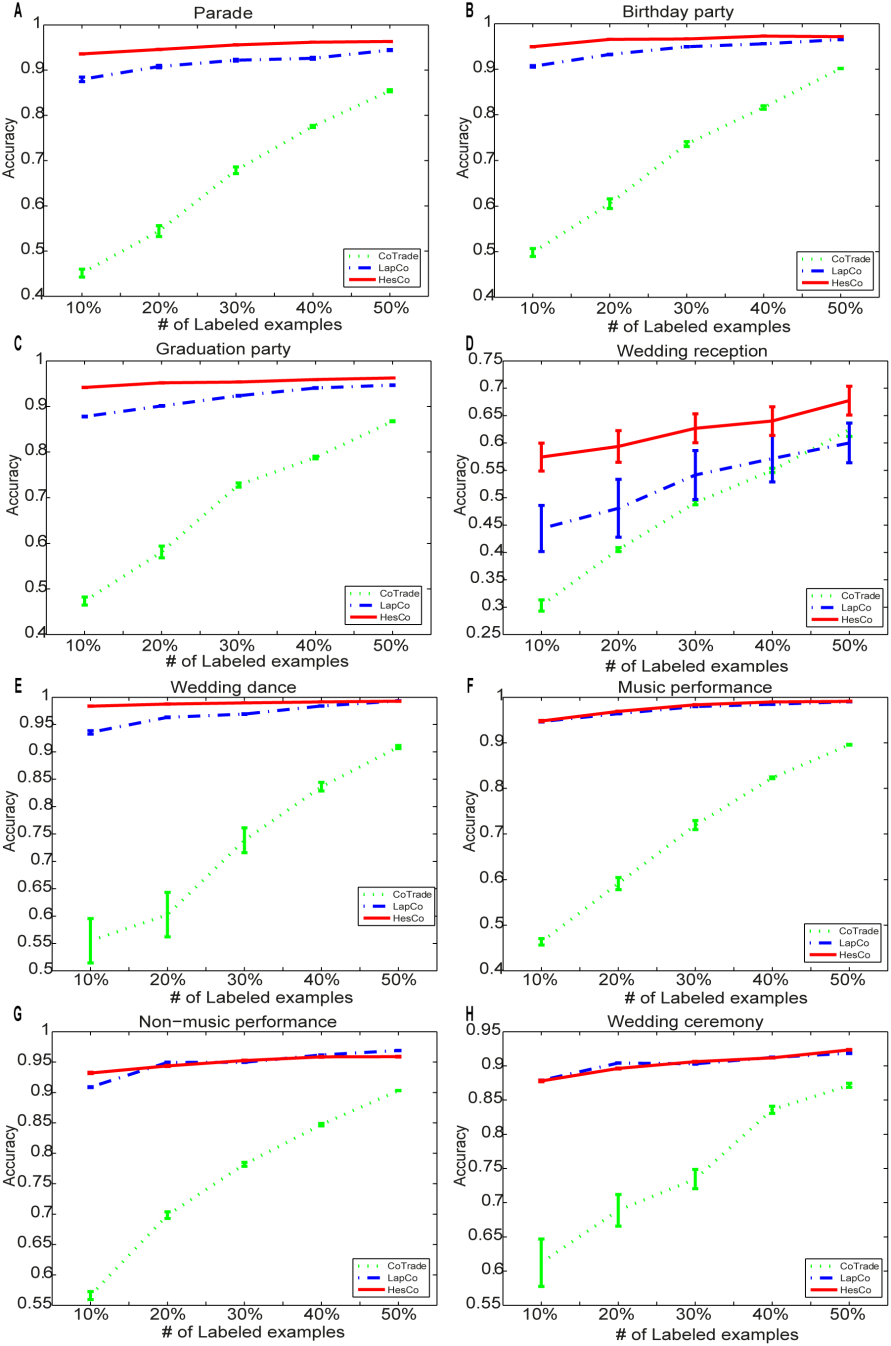
The accuracy of the different methods for the eight activity classes. Each subfigure corresponds to one activity class in the dataset. The x-coordinate is the number of labeled examples. (A) Parade. (B) Birthday party. (C) Graduation party. (D) Wedding reception. (E) Wedding dance. (F) Music performance. (G) Non-music performance. (H) Wedding ceremony.

Figure 5 shows the accuracy of the different methods for the eight activity classes. Each subfigure corresponds to one activity class in the dataset, and the x-coordinate is the number of labeled examples. Manifold regularized co-training methods, including LapCo and HesCo, significantly boost performance for every activity class, especially when the number of labeled examples is small. HesCo outperforms LapCo in most cases.

## Conclusion

Here we propose Hessian regularized co-training (HesCo) to boost co-training performance. In this method, each Hessian is first obtained from a particular view of examples. Second, Hessian regularization is used to explore the local geometry of the underlying manifold for the training of the classifier. Hessian regularization significantly boosts the performance of the learners and then improves the effectiveness of augmenting the training set in each co-training round. Comprehensive experiments on social activity recognition in the USAA dataset were conducted to evaluate the proposed HesCo algorithm, which demonstrated that HesCo outperforms baseline methods, including the traditional co-training algorithm and Laplacian regularized co-training, especially with small numbers of labeled examples.
